# Comparison of *PvLAP5* and *Pvs25* qRT-PCR assays for the detection of *Plasmodium vivax* gametocytes in field samples preserved at ambient temperature from remote malaria endemic regions of Panama

**DOI:** 10.1371/journal.pntd.0010327

**Published:** 2022-04-08

**Authors:** Nicanor Obaldía, Itza Barahona, José Lasso, Mario Avila, Mario Quijada, Marlon Nuñez, Matthias Marti

**Affiliations:** 1 Departamento de Investigaciones en Parasitología, Instituto Conmemorativo Gorgas de Estudios de la Salud, Panamá City, Panamá, Republic of Panamá; 2 Department of Immunology and Infectious Diseases, Harvard T.H. CHAN School of Public Health, Boston, Massachusetts, United States of America; 3 Wellcome Centre for Integrative Parasitology, Institute of Infection, Immunity and Inflammation, College of Medical, Veterinary & Life Sciences, University of Glasgow, Glasgow, Scotland, United Kingdom; 4 Departamento de Control de Vectores, Dirección General de Salud Pública, Ministerio de Salud de Panamá, Panamá, Republic of Panamá; Menzies School of Health Research, AUSTRALIA

## Abstract

**Background:**

As the elimination of malaria in Mesoamerica progresses, detection of *Plasmodium vivax* using light microscopy (LM) becomes more difficult. Highly sensitive molecular tools have been developed to help determine the hidden reservoir of malaria transmission in low transmission settings. In this study we compare the performance of *PvLAP5* and *Pvs25* qRT-PCR assays to LM for the detection of *Plasmodium vivax* gametocytes in field samples preserved at ambient temperature from malaria endemic regions of Panama.

**Methods:**

For this purpose, we collected a total of 83 malaria field samples during 2017-2020 preserved in RNAprotect (RNAp) of which 63 (76%) were confirmed *P*. *vivax* by LM and selected for further analysis. Additionally, 16 blood samples from local healthy malaria smear negative volunteers, as well as, from 15 malaria naïve lab-bred *Aotus* monkeys were used as controls. To optimize the assays, we first determined the minimum blood volume sufficient for detection of *PvLAP5* and *Pv18SrRNA* using *P*. *vivax* infected Aotus blood that was preserved in RNAp and kept either at ambient temperature for up to 8 days before freezing or was snap-frozen at -80° Celsius at the time of bleeding. We then compared the mean differences in gametocyte detection rates of both qRT-PCR assays to LM and performed a multivariate correlation analysis of study variables. Finally, we determined the sensitivity (Se) and specificity (Sp) of the assays at detecting gametocytes compared to LM.

**Results:**

Blood volume optimization indicated that a blood volume of at least 60 μL was sufficient for detection of *PvLAP5* and *Pv18SrRNA* and no significant differences were found between RNA storage conditions. Both *PvLAP5* and *Pvs25* qRT-PCR assays showed a 37-39% increase in gametocyte detection rate compared to LM respectively. Strong positive correlations were found between gametocytemia and parasitemia and both *PvLAP5* and *Pvs25* gametocyte markers. However, no significant differences were detected in the Se and Sp of the *Pvs25* and *PvLAP5* qRT-PCR assays, even though data from control samples suggested *Pvs25* to be more abundant than *PvLAP5*.

**Conclusions:**

This study shows that the *PvLAP5* qRT-PCR assay is as Se and Sp as the gold standard *Pvs25* assay and is at least 37% more sensitive than LM at detecting *P*. *vivax* gametocytes in field samples preserved in RNAp at ambient temperature from malaria endemic regions of Panama.

**Author summary:**

*Plasmodium vivax* is one of the five species of malaria (*P*. *falciparum*, *P*. *malariae*, *P*. *ovale* and *P*. *knowlesi)* that are transmitted to man by the bite of female anopheles mosquitoes. It causes ~14.3 million cases mainly in Southeast Asia, India, the Western Pacific and the Americas annually. In the Americas, malaria remains a major problem in underdeveloped areas and indigenous communities in the Amazon region and eastern Panama, where it is endemic and difficult to eliminate. As malaria elimination progresses, detection of *P*. *vivax* by light microscopy (LM) becomes more difficult. Therefore, highly sensitive molecular tools have been developed that use genetic markers for the parasite to help determine the hidden reservoir of malaria transmission. This study compares the performance of two molecular assays based on the genetic markers of mature gametocytes *PvLAP5* and *Pvs25* with LM. The study shows that the *PvLAP5* qRT-PCR assay is as sensitive and specific as the gold standard *Pvs25* assay and is at least 37% more sensitive than LM at detecting *P*. *vivax* gametocytes. These data suggest that the *PvLAP5* qRT-PCR assay can be a useful tool to help determine the hidden reservoir of transmission in endemic foci approaching elimination.

## Introduction

Each year an estimated 229 million cases and 409,000 deaths attributable to malaria mainly in children under 5 years are reported globally, 85% of which occur in Sub-Saharan Africa [1]. In other parts of the world malaria deaths occur mainly in non-immune individuals of all ages. The majority of malaria cases and deaths are due to *Plasmodium falciparum*, however in many regions outside of sub-Saharan Africa *P*. *vivax* predominates [1]. *P*. *vivax* is a major cause of morbidity and mortality in Southeast Asia, India, the Western Pacific and the Americas, and it remains present across sub-Saharan Africa [2]. The global *P*. *vivax* burden is estimated at 14.3 million cases per year [3]. In the Americas, malaria continues to be a major problem in poorly developed areas and indigenous communities such as part of the Amazon region, Eastern Panama [4,5] and the Darien gap [6], while it is under control in urban settings [7,8].

Global efforts to eradicate malaria have been stimulated by a dramatic drop in the incidence of the disease in sub-Saharan Africa [9–11]. For instance, between 2000 and 2015 the incidence of malaria declined by approximately 37% and the death rate by 60% worldwide [12]. Similarly, the global burden of *P*. *vivax* malaria decreased by 41.6% between 2000 and 2017, and in the Americas by 56.8% since 2000 [13]. Unfortunately, parasite resistance to the major anti-malarial drugs including Artemisinin is rapidly spreading and threatening ongoing elimination strategies in the Americas and elsewhere [14–18].

Major gaps in our understanding of *P*. *vivax* biology, pathogenesis and epidemiology remain [19,20]. In addition, little is known about its population structure in many endemic regions [6], the extent of asymptomatic carriers [19,21], and the role played in transmission by cryptic reservoirs such as the bone marrow [21,22] and spleen [23]. Many experts agree that *P*. *vivax* will persist after *P*. *falciparum* is eliminated, due to the existence of latent liver stages (hypnozoites) that can cause relapses even years after infection [19]. On the other hand, there is currently no system of continuous *in vitro* culture that would accelerate basic research and development of new drugs, vaccines, and diagnostic tests [24,25]. Therefore, the conventional diagnostic tools that have supported the epidemiological and clinical understanding of vivax malaria may not be adequate for studying the complex biology and epidemiology of this parasite in low transmission settings [3].

Recent studies have reported high rates of sub-microscopic *P*. *vivax* infections in areas of low transmission such as the Solomon Islands [26]. Similar conditions are found in endemic remote regions of Panama, where its inhabitants live in low transmission settings mostly associated with Amerindian reservations [8]. Such settings contain multiple foci or pockets (“Hot Spots”) of transmission, which can present logistical and technical challenges for malaria control programs due to their remoteness and limited sensitivity of available diagnostic tests (i.e., thick blood smears and rapid diagnostic tests (RDTs) [3,6,27].

*P*. *vivax* presents differences in biological features compared to *P*. *falciparum* [3]. For instance, gametocytes of *P*. *vivax* appear early in infection, between 3-5 days after the first asexual parasites are detected in circulation, and before the patient is symptomatic [28], while *P*. *falciparum* gametocytes appeared much later. Intriguingly, *P*. *vivax* gametocytes appear to have a half-life of one day, with a maximum circulation time that has been estimated at three days, with male gametocytes having a shorter lifespan than female gametocytes [29]. Hence, *P*. *vivax* can be transmitted to mosquitoes even before the onset of symptoms [19,30,31]. The reason for the early transmissibility is the relatively short gametocyte development of approximately 48 hours [21] compared to 10-12 days in *P*. *falciparum*. As in *P*. *falciparum*, developing (immature) *P*. *vivax* gametocytes are predominantly found in the hematopoietic niche of the bone marrow and possibly spleen [21,22,32]. This hidden reservoir of *P*. *vivax* parasites that has only recently been elucidated, fundamentally changes existing paradigms of *P*. *vivax* biology, pathogenesis and epidemiology [3,33].

The detection of mature *P*. *vivax* gametocytes in blood samples by light microscopy (LM) is imprecise due to their low levels in circulation [34], about 2-6% of the total parasitemia, but previous studies suggest that *P*. *vivax* gametocytemia and parasitemia are strongly correlated [29,35].

Molecular diagnostic tools that detect asymptomatic *P*. *vivax* carriers with sub patent infections have been developed [27,36]. These assays use primers targeting the *P*. *vivax* 18s ribosomal RNA gene (*Pv18SrRNA*) and *Pvs25* (PVX_111175), a gene encoding a mature gametocyte marker and ookinete surface antigen located on chromosome 6 and ortholog of *P*. *falciparum Pfs2*5 (PF3D7_103100) [34,37]. Both genes lack introns and can be amplified from gDNA.

We recently characterized *PvLAP5* (PVX_117900), a novel *P*. *vivax* gene encoding a mature gametocyte surface marker LCCL domain containing protein, located on chromosome 12 and ortholog of *P*. *falciparum PfFNPA* (PF3D7_1451600; PF14_0491). Importantly, this gene contains 2 exons flanking an intron allowing the development of a qRT-PCR assay that uses exon-exon spanning primers, therefore preventing gene amplification from contaminating gDNA [4,21]. Of note, until now there are no published studies detailing a gametocyte time course for *P*. *vivax* or *P*. *falciparum* that could provide high resolution data on copy numbers and/or expression dynamics during gametocyte development for these markers. However, *PvLAP5* and *Pvs25* transcripts levels are highly correlated in samples collected 48h after initiating *P*. *vivax ex vivo* cultures, and at similar levels to those measured in blood samples directly drawn from infected *Aotus* monkeys. These data are in agreement with their gametocyte-specific patterns in *ex vivo* microarrays [21].

Other *P*. *vivax* gametocyte markers such as *Pvs28*, *Pv41*, *Pvs48/45*, and *Pvs230* have been described and characterized as well [38–42], and are candidate antigens for the development of transmission blocking vaccines [43].

Molecular diagnostic methods play an important role in malaria elimination programs to determine with greater precision the transmission reservoir and design interventions tailored to endemic areas of low transmission [44]. Moreover, detection and determination of gametocyte densities has been considered an important metric for evaluating the effectiveness of antimalarial interventions to reduce transmission [45]. Understanding gametocyte carriage in low transmission settings “Hot Spots”, would contribute to further elucidate transmission patterns and the epidemiology of the disease, essential steps for developing malaria control strategies and accelerate towards elimination [46].

This study describes the field performance of a *PvLAP5* qRT-PCR assay compared to *Pvs25* (gold standard) and LM, for the detection of *P*. *vivax* gametocytes on clinical samples preserved at ambient temperature in RNAp from malaria endemic regions of low transmission in Panamá. The study constitutes a first step for the implementation of a large-scale malaria molecular epidemiological survey to determine the hidden reservoir of transmission in the country.

## Materials and methods

### Ethics statement

Study protocol and consent form approval was obtained from The Gorgas Memorial Institutional Bioethics Review Committee (No. 276/CBI/ICGES/16). Written informed consent was obtained from the participants. Animal blood samples used in this study were obtained from the ICGES malaria strains repository, or from animals inoculated for use as donors in other protocols. Collection of malaria naïve monkey blood was carried out as part of a routine animal health program. All animals were maintained and treated in accordance with the Guide for the Care and Use of Laboratory Animals, eighth edition 2011, National Research Council, Washington, DC.

### Study design

A prospective cross-sectional study was implemented between 2017-2020 to determine the performance of a PvLAP5 qRT-PCR assay for the detection of *P. vivax* gametocytes, using blood samples collected from *P*. *vivax* malaria positive volunteers detected by technicians from the National Vector Control Department (NVCD) of the Ministry of Health (MINSA) of Panamá.

### Study site

**Fig 1:**
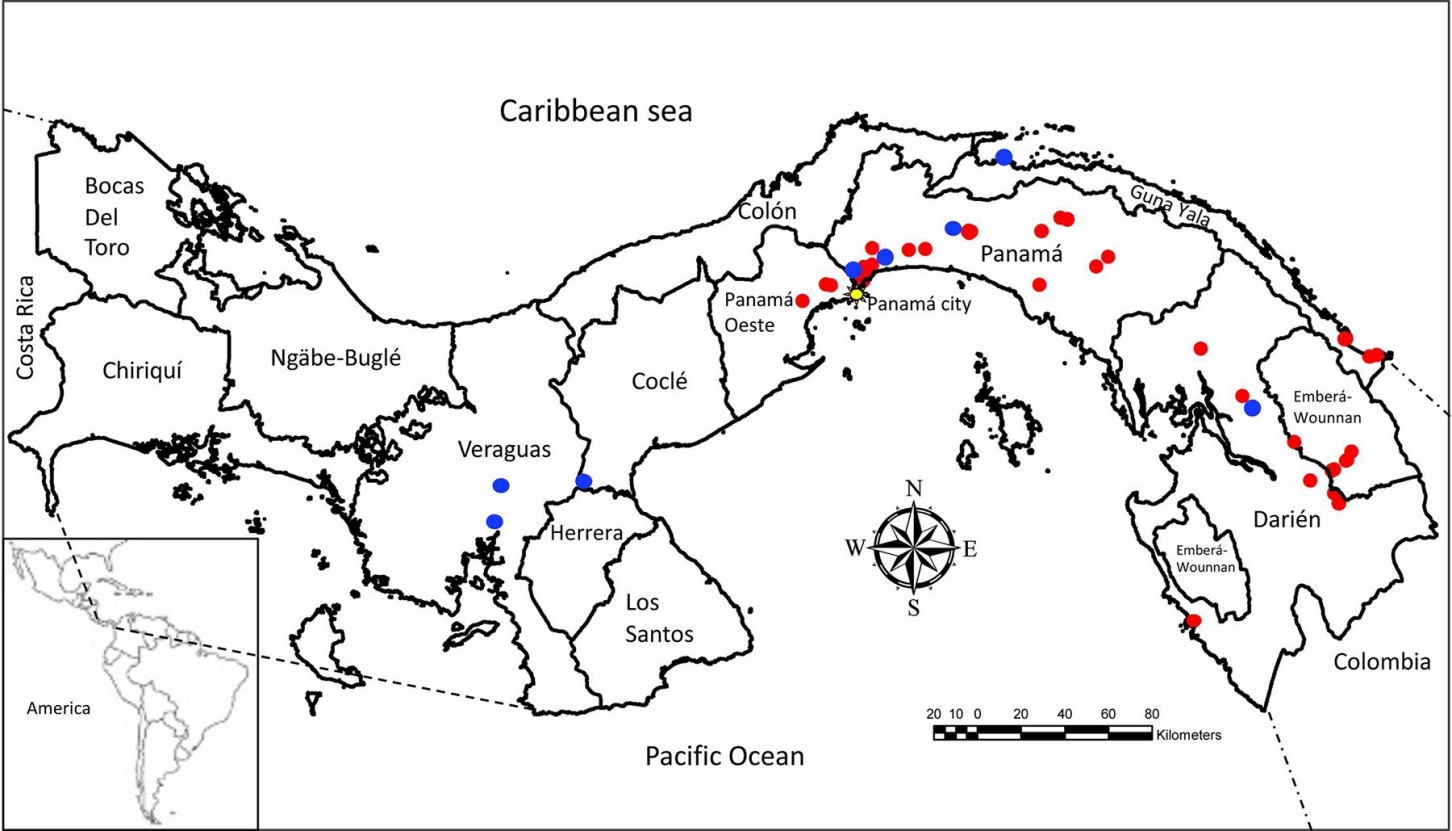
*Plasmodium vivax* samples collection sites. Map of Panama by province showing location of samples collection sites during 2017-2020. Red dots indicate *P*. *vivax* samples collection sites where one or more samples were collected. Blue dots indicate healthy control sample collection sites. Inset indicates location of Panama in the American continent. Base map downloaded from www.gadm.org under licence CC-BY. www.gadm.org/maps/PAN_1.html.

The study was conducted in the Republic of Panamá located in Central America between 7° and 10° north latitude and 77° and 83° west longitude, bordering Colombia to the east and Costa Rica to the west, the Caribbean Sea to the north and the Pacific Ocean to the South, with a total land area of 74,340 Km^2^ and a population of 3,657,024 (July 2015 est.) (**Fig 1**). To delimit and select the study collection sites, we first prepared a *P*. *vivax* case incidence map at the level of corregimiento (smallest political division) using base maps downloaded from gadm.org under licence CC-BY and with data obtained from the NVCD of the Ministry of Health of Panama, using the ArcMap 10.6.1. software (Esri, Redlands, CA). NVCD field technicians from selected high incidence endemic areas were trained in the collection of malaria field samples for molecular studies (**Fig Aa in S1 File**).

### Characteristics of the study population

Study participants comprised of volunteers that were residents of the provinces of Darien, Panama, Panama oeste and the Comarca of Guna Yala (**Fig 1)**. In total 83 malaria smear positive samples were submitted for analysis. In addition, 16 malaria smear negative samples were collected from healthy volunteers living in the provinces of Coclé, Darien, Herrera, Panama, Veraguas and the Comarca Guna Yala, and from 15 malaria naïve lab-bred *Aotus* monkeys from the Gorgas Memorial Institute *Aotus* colony. These controls serve as negative controls for determination of the assays positive/negative threshold, totalizing 31 malaria smear negative samples. Geographic, demographic, and socioeconomic information of study participant was collected using an epidemiological survey form developed with the Survey123 for ArcGIS online survey software (Esri, Redlands, CA). Geographic coordinates were taken from the centre town of the place of residence to avoid identification of volunteers. Demographic data including gender (males or female), age (years) and ethnicity, as well as, socioeconomic determinants such as type of house, number of family members per household, literacy and employment status were also collected.

### Blood sampling

Thin and thick blood smears were prepared from a finger-prick made with a lancet, air-dried, and transported to the laboratory for staining. Between 60 and 120 μL of finger prick blood was collected into 1.8 ml NUNC cryovials containing 500 μL of RNAprotect (RNAp) (Qiagen, Germany) for RNA isolation and qRT-PCR assay. Samples were transported at ambient temperature and the cryovials were stored at -80 C upon arrival to the laboratory.

#### Microscopy

Giemsa stained thick and thin blood smears were examined by LM for species identification, stage differential count and parasite density determination. Parasitemia was determined by quantifying the number of malaria infected red blood cells (iRBCs) among 500 – 2000 RBCs on a thin blood smear and expressing the results as % parasitemia (% parasitemia = parasitized RBCs/total RBCs) x 100), or quantifying parasites against white blood cells (WBCs) on the thick smear until 500 or 1000 WBCs were counted (parasitized RBCs x μL of blood, assuming 8,000 WBC/μL of blood). Stage differential counts were expressed as percentage of total parasite stages counted.

### qRT-PCR assay

#### Parasites

*P*. *vivax* SAL-1 infected anticoagulated whole blood obtained from experimentally inoculated and malaria naïve *Aotus* monkeys kept at the Gorgas Memorial Institute in Panama, were used as positive and negative controls for the qRT-PCR assay as described [4]. To determine the cut-off point Cycle Threshold (Ct) value of the qRT-PCR assays, fifteen male and female monkeys were used as negative controls. *P*. *vivax* SAL-1 infected anticoagulated (Sodium Citrate 4% Solution, Sigma, St. Louis, MO) whole blood obtained from a donor monkey (MN12939) was used as positive control.

#### Primers

We used forward and reverse primers sets for *PvLAP5*, *Pvs25* and *Pv18SrRNA* as previously described [4] (**Table A in S1 file**). *PvLAP5* primers were designed to span exon-exon junctions to minimize amplification from gDNA. As gold standard control, we used primers for the gametocyte marker *Pvs25*. Primer sets including *PvLAP5*, *Pvs25* and *Pv18SrRNA* were synthesized by Genscript (Piscataway, NJ, USA).

#### RNA extraction and cDNA synthesis

RNA was isolated from RNAp preserved blood samples using the Qiagen RNAeasy Plus kit that includes a gDNA eliminator column (Qiagen, Germany) per the manufacturer’s instructions. After determination of the RNA concentration using a NanoDrop ND spectrophotometer (Thermo Fisher Scientific Inc, USA), the isolated nucleic acid was treated to remove residual DNA with a DNA-free kit (Ambion, Life Technologies, USA). The treated RNA was then transcribed to cDNA with the QuantiTect Reverse Transcription Kit (Qiagen, Germany) following the manufacturer’s instructions.

#### Procedure for the qRT-PCR assay

Assay reactions were performed in a QuantStudio 5 Real-Time PCR 384 well plate system (Applied Biosystems, USA) as described [21]. Each Fast SYBR Green reaction (final volume of 20 μL) consisted of Master Mix Fast SYBR Green (Applied Biosystems, USA), forward and reverse primers mix at 300 nM concentration and 2 μL of cDNA. Thermal cycle conditions were as follows: 10 min at 95°C followed by 40 cycles at 95°C for 15 s, 60°C for 1 min. A melting curve analysis was added at the end of the reaction cycle to determine the specificity of the reaction or the generation of an unspecific signal due to the formation of primer dimers [47]. Samples were analysed in duplicate. Each plate included a positive and negative control (uninfected sample) and a negative amplification control without RT enzyme to exclude false positives due to the presence of genomic DNA. A Ct value of ≤ 38 for the endogenous *Pv18SrRNA* gene marker was used as the positive threshold for *P*. *vivax* detection. The negative cut-off point Ct value was calculated from the geometric mean (GM) Ct values of sixteen malaria smear negative healthy human volunteers and fifteen malaria naïve monkey controls as shown on **Tables B and C in S1 File**.

### Performance of the qRT-PCR assay

#### qRT-PCR assay of field samples

To validate the qRT-PCR assay and sample preservation system in the field, we first determined the mean negative Ct value threshold using 16 smear negative samples for each marker. We subsequently tested 63 smear positive *P*. *vivax* samples out of 83 samples submitted for *PvLAP5*, *Pvs25* and *Pv18SrRNA* transcripts as described [21]. Only positivity (Ct ≤ 38) but not copy numbers were reported for *PvLAP5*, *Pvs25* and *Pv18SrRNA*. Representative qRT-PCR assay amplification and melt curve plots of two microscopic positive *P*. *vivax* samples done in triplicate are shown in **Fig B in S1 File**.

#### Assay validation

Using the open web based tool “Diagnostic Test Evaluation Calculator” (https://www.medcalc.org/calc/diagnostic_test.php) (MedCalc Software Ltd, Osten, Belgium) we determined the following parameters: i) the sensitivity (Se), or the probability that a test result will be positive when the disease is present (true positive rate); ii) the specificity (Sp), or the probability that a test result will be negative when the disease is not present (true negative rate); iii) the positive likelihood ratio (PLR), or the ratio between the probability of a positive test result given the presence of the disease and the probability of a positive test result given the absence of the disease (True positive rate /False positive rate = Sensitivity/ (1-Specificity)); iv) the negative likelihood ratio (NLR), or the ratio between the probability of a negative test result given the presence of the disease and the probability of a negative test result given the absence of the disease (False negative rate/True negative rate = (1-Sensitivity) /Specificity)); v) the positive predictive value (PPV), or the probability that the disease is present when the test is positive; and vi) the negative predictive value (NPV), or the probability that the disease is not present when the test is negative. These two last definitions depend on the disease prevalence [48,49].

The data was then tabulated on a series of 2 x 2 tables as follows: a) the number of *P*. *vivax* gametocytes positive smears (disease present) coded 1; b) number of gametocyte negative smears (disease absent) coded 0; c) the number of qRT-PCR positive samples (test positive) Ct value ≤ 38 and d) number of qRT-PCR negative samples (test negative) Ct value > 38 (test negative) for each gametocyte gene marker (*PvLAP5* and *Pvs25*) and for performance of the *Pv18SrRNA* endogenous marker we used the smear positive detection proportion [50–52]. For validation we calculated the theoretical minimum number of positive and negative samples necessary to achieve a level of sensitivity of 97% and specificity of 99% with a margin of error of 2-5% and a confidence level of 95% as described [51].

#### Statistics

Statistical analysis was done using the statistical and graphics software Prism 6.0 (GraphPad Software, Inc, La Jolla, CA, USA), the JMP Pro Statistical software (SAS Institute Inc., Cary, NC, USA) and the Web based Diagnostic Test Evaluation Calculator (https://www.medcalc.org/calc/diagnostic_test.php) (MedCalc Software Ltd, Osten, Belgium).

## Results

The overall goal of this study was to determine the performance of a qRT-PCR assay for the detection of the *P*. *vivax* gametocyte marker *PvLAP5* using clinical samples preserved in RNAp at ambient temperature from remote areas of Panama, compared to *Pvs25* (gold standard) and LM. Specifically, we aimed to: i) compare the detection of *P*. *vivax* gametocytes by LM to qRT-PCR assays *PvLAP5*, and *Pvs25*, and ii) validate the assay protocol for ongoing elimination efforts in Panama.

### *Plasmodium vivax* in Panama during 2017-2020

To contextualize the study by person, place and time, using the NVCD malaria data for the years 2017-2020, we prepared a case incidence map by 10,000 population at the corregimiento level (smallest political division) using ArcMap 10.6.1 (ArcGIS, Esri, Redlands, CA), and epidemiological curves stratified by year, month, and age groups, as well as, a pie chart of the ethnic distribution of cases for the years 2017-2019. As depicted in **Fig Aa in S1 File**, the highest *P*. *vivax* incidence occurred in individuals living in the indigenous comarcas of Guna Yala and Embera-Wounan in the province of Darien with 315-2,176 cases per 10,000 population, and in the comarcas of Madugandi and Wargandi in the provinces of Panama and Darien, with 2,177-4,177 cases per 10,000 population. The majority of individuals were less than 29 years old (**Fig Ab and Ac in S1 File**), and of Amerindian ethnicity (**Fig Ad in S1 File**). The epidemic curve for the years 2017-2020 shows malaria cases peaking in February during the middle of the dry season that runs from December to April, and again in December at the beginning of the next dry season (**Fig Ac in S1 File**).

It should be noted that during 2019 the number of cases had a 43% increase compared to the previous year, with a peak of more than 300 cases reported during the dry season and a similar trend in 2020, increase that might have been the result of the recent introduction of RDTs as a field diagnostic tool in 2017 (**Fig Ab in S1 File**), although we cannot rule out other causes such as environmental factors [53], or an unprecedented increase in continental and extracontinental migrants in transit from South America which reached a peak of 134,000 in 2021 up from 8,000 in 2020 [54].

### Characteristics of the study population

Study participants comprised of volunteers that were residents of the provinces of Darien, Panama, Panama oeste, and the Indigenous Comarca of Guna Yala (**Fig 1)**. Of the total of 83 samples submitted, 38/83 (46%) were collected in Darien, 26/83 (31%) in the provinces of Panama and Panama oeste and 19/83 (23%) in the comarca Guna Yala. Of these, 9/83 (11%) were diagnosed as *P*. *falciparum*, and 11/83 (13%) were excluded because they were either malaria negative by microscopy, the smears were broken or unreadable or there was insufficient sample volume.

Among the 63 *P*. *vivax* samples selected for evaluation, 26 (41%) were collected in the province of Darien, 24 (38%) in Panama and Panama oeste, and 13 (21%) in the comarca Guna Yala. Twenty-four of the samples (38%) were collected from females with a median age of 22 (min, max) (0.5, 74) years old, and 39 (62%) from males with a median age of 28 (2, 76) years old. Analysis of the survey taken to 73/83 volunteers, showed that 42% were ethnic Amerindians, 26% reported to be unemployed, 14% were illiterate, and 44% lived in a type 2 or 3 house (known as rancho or bohío, constructed with a wooden frame cover with royal palm leaves and other deciduous materials) as defined elsewhere [8], with 6 dwellers on average per household (**Tables D and E in S1 File).**

Additionally, 16 malaria smear negative samples were collected from local healthy volunteers residents of the provinces of Cocle, Darien, Panama, Panama oeste, and Veraguas (**Fig 1**). These controls were 10 males with a median age of 59 (38, 65) years old, and 6 females with a median age of 25 (15, 46) years old (**Table B in S1 File**). Additional controls were 15 malaria naïve adult (> 2 years old) lab-bred *Aotus* monkeys from the Gorgas Memorial Institute *Aotus* colony (**Table C in S1 File**), to serve as negative controls and for determination of the negative cut-off point Ct value. In total, we used 31 malaria smear negative samples.

### Parasite characteristics by light microscopy

**Fig 2:**
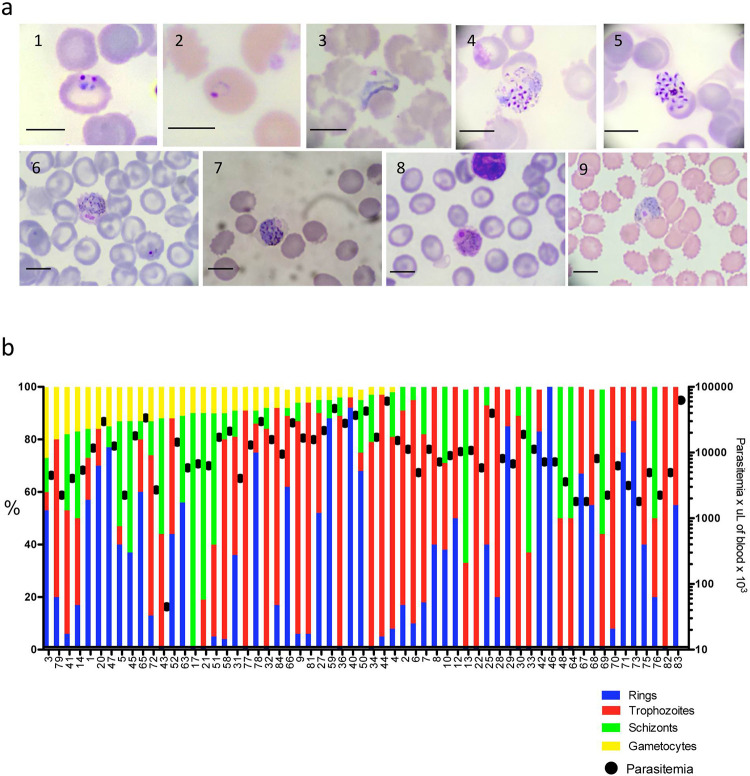
Microscopic detection of *Plasmodium vivax* asexual and sexual stages. a) Asexual and sexual stages of selected field samples: 1-2) Rings; 3) Trophozoite; 4-5) Schizonts; 6-9) Gametocytes. Giemsa stain. Black bar = 8 μm. b) Percent parasite stages and parasitemia of selected field samples. N = 61.

To determine the proportion of asexual and sexual stages we examined Giemsa thin blood smears from each sample. Representative images of *P*. *vivax* asexual and sexual stages are shown in **Fig 2A**.

Parasite stages were detected at similar levels in the 63 *P*. *vivax* positive smears examined except for the less abundant gametocytes that were detected in only 35/63 (55.5%) and were significantly different than the proportion of rings 48/63 (76%) (*X*^2^ (1, *N* = 63) = 5.966, *p* = .00146) and trophozoites 57/63 (90%) (*X*^2^ (1, *N* = 63) = 19.50, *p* < .00001). Schizonts were detected in 40/63 (63%) (*X*^2^ (1, *N* = 63) = 0.8235, *p* = .03642), (**Fig 2B**).

As previously reported schizont and gametocyte stages were present at significantly lower levels in the peripheral blood than rings and trophozoites, presumably due to their tissue enrichment as it has been reported to occur in the bone marrow and spleen [21,23].

### Optimization of blood volume and sample preservation conditions for detection of *P*. *vivax* by qRT-PCR

To optimize the blood volume and processing of field samples for parasite stage analysis by qRT-PCR, we designed an experiment simulating field conditions with the assumption that the field samples were going to be in transit to the laboratory for an average of 8 days. For this purpose, we first amplified the reference strain *P*. *vivax* SAL-1 in the Aotus non-human primate (NHP) model. Fifteen days after infection, when parasitemia reached 51,080 parasites x μL, citrated anticoagulated blood was collected in RNAp. A total volume of 60 or 120 μL of *P*. *vivax*-infected blood, respectively, was preserved in 500 μL of RNAp and snap-frozen immediately at -80°C or kept for eight days at ambient temperature (~ 27°C) until freezing for further analysis. Samples across conditions were then processed for RNA isolation and subsequent cDNA synthesis and qRT-PCR. Of note, this experiment did not aim to exhaustively test different preservation conditions. Comparison using two-way ANOVA revealed no statistically significant differences across conditions using *PvLAP5* and *Pv18SrRNA* (**Table 1 and Fig C in S1 File**). Nonetheless, the Ct values of *Pv18SrRNA* and *PvLAP5* obtained from ambient temperature stored samples were 2-3 cycles higher than the Ct values obtained from samples stored at -80° C, most notable when 120 μL samples were used. These results suggest a 4-8-fold lower quantity of RNA in samples stored at ambient temperature compared to those stored at -80° C.

**Table 1:** qRT-PCR Ct values of two blood volumes of *Plasmodium vivax* infected blood preserved with RNAprotect and kept at ambient temperature for eight days or snap-freeze at – 80° Celsius.

	Ct value							
	Ambient temperature for 8 days and freeze				Snap-freeze -80°C			
	*PvLAP5*		*Pv18SrRNA*		*PvLAP5*		*Pv18SrRNA*	
Blood volume	Mean (SEM)	n	Mean (SEM)	n	Mean (SEM)	n	Mean (SEM)	n
60 μL	35 (0.7) ns	3	32 (2.3) ns	3	33 (1.8) ns	4	32 (3.8) ns	4
120 μL	33 (1.1) ns	2	28 (1.3) ns	2	31 (0.8) ns	5	25 (2.7) ns	4

SEM = Standard error of the mean

n = number of experimental replicates

Ct values ≤ 38 are considered positive

ns = non-significant (two-way ANOVA)

Blood volume optimization indicated that a volume of at least 60 μL was sufficient for detection of *PvLAP5* and *Pv18SrRNA*, and no significant difference was found between blood kept at room temperature in RNAp for 8 days and snap-freeze at -80° C, or snap-freeze upon collection to stop degradation (**Table 1**). Therefore, we decided to collect a minimum of 60 μL of sample in 500 uL of RNAp that were kept at ambient temperature during transit and frozen at -80 C upon arrival at the laboratory.

### *PvLAP5* is as sensitive and specific as *Pvs25* at detecting *P*. *vivax* gametocytes in field clinical samples preserved in RNAp

**Fig 3:**
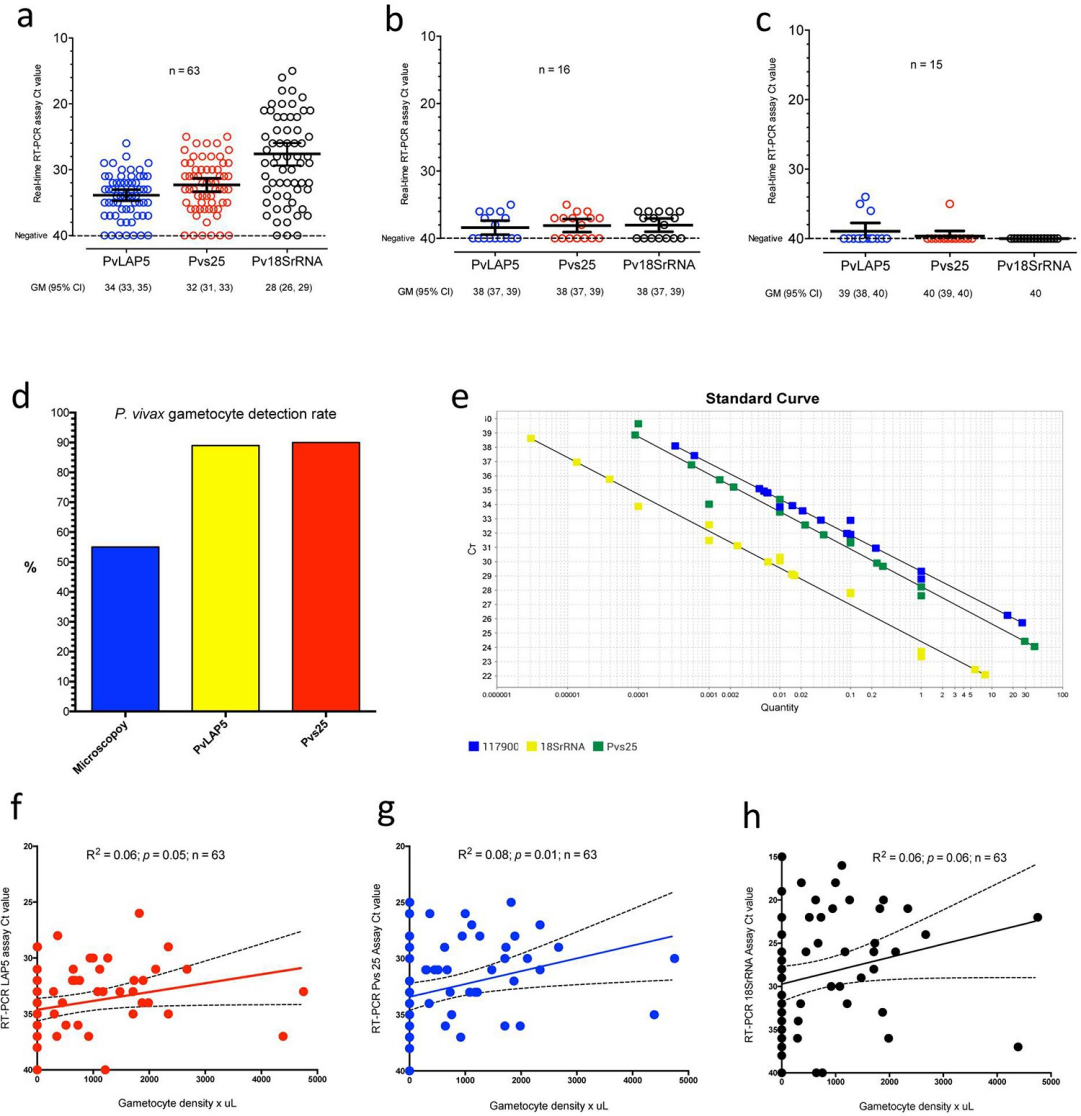
Molecular detection of *Plasmodium vivax*. a) qRT-PCR Ct values of *P*. *vivax* positive field samples for gametocyte markers *PvLAP5* and *Pvs25* and constitutive marker *Pv18SrRNA*. N = 63. b) qRT-PCR Ct values of *P*. *vivax* negative human control samples for gametocyte markers *PvLAP5* and *Pvs25* and constitutive marker *Pv18SrRNA*. N = 16. c) qRT-PCR Ct values of *P*. *vivax* malaria naïve lab-bred *Aotus* monkeys samples for gametocyte markers *PvLAP5* and *Pvs25* and constitutive marker *Pv18SrRNA*. N = 15. d) *P*. *vivax* gametocyte detection rate plot. Microscopy vs qRT-PCR *PvLAP5* and *Pvs25* assays. N = 63. e) qRT-PCR standard curve plots of gametocyte markers *PvLAP5* and *Pvs25* and constitutive marker *Pv18SrRNA*. f-h) Linear regression plots of gametocyte markers *PvLAP5 and Pvs25* and constitutive marker *Pv18SrRNA* against gametocyte density x μL. N = 63. *R*^*2*^ = coefficient of determination. Dotted lines represent 95% confidence intervals. Ct values ≤ 38 are considered positive.

We successfully performed qRT-PCR molecular assays on the subset of 63 *P*. *vivax* smear positive field cases, 16 smear negative samples from healthy volunteers, and 15 malaria naïve *Aotus* monkeys (**Fig 3A, 3B and 3C**). The theoretical minimum number of positive and negative samples needed for a level of sensitivity of 97% and specificity of 99% with a margin of error of 2-5% and a confidence level of 95% was estimated in ~ 45 positive and ~ 15 negatives samples [51]. Of note is that ninety-eighth percent (62/63) of the samples tested spent a median of 7 (0, 192) days in transit before freezing (**Table F in S1 File**).

The assay detection rate for all *P*. *vivax* stages in positive smears using the constitutive marker *Pv18SrRNA* was 93.6% (59/63) and for sexual stage markers it was 90.4% (57/63) for *Pvs25* and 88.8% (56/63) for *PvLAP5*. Based on the detection rate for each marker a sensitivity pattern emerged as follows: *Pv18SrRNA > Pvs25 > PvLAP5*. However, the proportion of gametocytes detected did not differ between the two sexual markers (*X*^2^ (1, *N* = 63) = 0.085, *p* = 0.7696). In contrast, microscopic examination detected gametocytes in only 55.5% (35/63) of the *P*. *vivax* positive smears examined (**Fig 3D**). Interestingly, several Ct values out of bound from the upper limit of the 95% Confidence Interval (CI) were detected by Melting Curve Analysis in the control samples and attributed to spurious signal emitted by primer dimers (**Fig 3B and 3C** and **Figs D and E in S1 File)**.

We then used serial limiting dilutions of a known positive clinical sample (i.e., previously determined parasite stage concentration) and established the clinical limit of detection (LOD) of the qRT-PCR assay at 1.44 gametocytes x μL for *PvLAP5* and 0.144 gametocytes x μL for *Pvs25* (**Fig 3E**). Thus, the *PvLAP5* and *Pvs25* qRT-PCR assays were estimated to be 5-50 fold more sensitive than the theoretical qRT-PCR LOD that had been previously reported at 9.6 gametocytes x μL for *Pvs25* [55]. This assumes that the detection threshold of the qRT-PCR assay was only limited by the amount of blood subject to amplification.

To further compare the detection rate of the qRT-PCR assays to microscopy (the gold standard for gametocyte detection), we first divided the samples into two groups based on their gametocyte detection by microscopy (negative = 0 and positive = 1). We used a multiple t-test and determined the difference in means between groups for study variables: age, sample days to laboratory, RNA concentration in ng/μL, mean parasitemia %, parasitemia density x μL and Ct values for *PvLAP5*, *Pvs25*, and *Pv18SrRNA*. Results of the analysis showed a statistically significant difference in the means between groups, for variables mean parasitaemia % (*p* = 0.0199) and parasitemia density x μL (*p* = 0.0261), suggesting that detection of gametocytes was associated with parasitaemia level. Moreover, a statistically significant difference was found between groups for all qRT-PCR assays (*PvLAP5*: *p* = 0.0201, *Pvs25*: *p* = 0.00006; *Pv18SrRNA*: *p* = 0.0139), but not for variables age (*p* = 0.6804), days to laboratory (*p* = 0.2418) or RNA concentration (*p* = 0.5449), suggesting that detection of *P*. *vivax* parasites was not associated with the variable time spent in transit to the laboratory, nor with the RNA concentration of the samples (**Table 2**).

**Table 2:** Summary statistics of *Plasmodium vivax* grouped by microscopic detection of gametocytes.

	Gametocyte microscopy detection		
	0	1	*p value*
**Age (years)**			
Mean (SD)	32 (22)	29 (24)	0.6804
N	21	29	
**Sample days in transit**			
Mean (SD)	24 (49)	12 (33)	0.2418
N	28	34	
**RNA ng/μL**			
Mean (SD)	11.08 (15)	9.3 (11.63)	0.5449
N	27	34	
**Parasitemia %**			
Mean (SD)	0.21 (0.28)	0.39 (0.31)	**0.0199**
N	28	35	
**Parastiemia x μL**			
Mean (SD)	9,852 (12,675)	17,654 (14,118)	**0.0261**
N	28	35	
***PvLAP5* (Ct value)**			
Mean (SD)	35.35 (3.18)	32.97 (3.12)	**0.0201**
N	28	35	
***Pvs25* (Ct value)**			
Mean (SD)	34.71 (3.91)	30.82 (3.27)	**0.00006**
N	28	35	
***Pv18SrRNA* (Ct value)**			
Mean (SD)	30.85 (5.77)	26.74 (6.86)	**0.0139**
N	28	35	

*p* value **=** multiple t-test, alpha = 5%, Sidak-Bonferroni method

SD = standard deviation

0 = negative

1 = positive

Further examination using a multivariate analysis approach revealed a significant negative correlation between age and days in transit (r = -0.3059, p < 0.01) with no apparent clinical significance. In contrast we observed a significant positive correlation between parasitemia density and asexual stages (r = 0.9982, p < 0.001), and a moderate positive correlation with gametocytaemia (r = 0.5205, p < 0.001), indicating that as one variable increases the other one increases in a monotonic fashion [56]. These results confirm past observations that gametocyemia mirrors parasitemia [29]. Similarly, a moderate negative correlation was found between parasitemia and gametocyte markers *PvLAP5* (*r = -*0.5370, p < 0.001) and *Pvs25* (r = -0.5137, p < 0.001), indicating that as parasitemia increases gametocyte marker Ct values decreases in a monotonic fashion [56]. Likewise, a significant positive correlation was detected between gametocyte markers *PvLAP5* and *Pvs25* (r = 0.8507; p < 0.001) and *PvLAP5* and *Pv18SrRNA* (r = 0.7533; *p* < 0.001), substantiating this finding (**Table 3**). Indeed, regression analysis showed a significant association between gametocytemia and qRT-PCR Ct values for gametocyte markers *PvLAP5* (R^2^ = 0.06, *p* = 0.05) and *Pvs25* (R^2^ = 0.08, *p* = 0.01) and constitutive marker *Pv18SrRNA* (R^2^ = 0.06, *p* = 0.06), with only 6-8% of the variance accounted for the independent variable gametocytemia, and 92-94% remaining unexplained (**Fig 3F, 3G and 3H)** [49].

**Table 3:** Multivariate analysis of *Plasmodium vivax* field cases.

Strength of correlation								
Variables	Age	Days in transit	Parasitemia density	Asexuals x *μ*L	Gametocytemia x μL	*PvLAP5*	*Pvs25*	*Pv18SrRNA*
Age	1	**-0.3059** *	-0.1668	-0.1701	-0.0321	0.2495	0.2876	0.1965
Days in transit		1	-0.007	-0.0082	0.0136	-0.0236	-0.0337	-0.1827
Parasitemia density			1	**0.9982** ***	**0.5205** ***	**-0.5370** ***	**-0.5137** ***	**-0.4799** ***
Asexuals x μL				1	**0.4684** ***	**-0.5286** ***	**-0.5006** ***	**-0.4646** ***
Gametocytemia x μL					1	**-0.3850** ***	**-0.4410** ***	**-0.4548** ***
*PvLAP5*						1	**0.8507** ***	**0.7553** ***
*Pvs25*							1	**0.8891** ***
*Pv18SrRNA*								1

*p* values for each comparison = *< 0.05; **< 0.01; ***< 0.001

N = 60

To assess the performance of the qRT-PCR assays at detecting asexual and sexual stages in *P*. *vivax* smear positive field samples preserved in RNAp at ambient temperature, we determined the Se, Sp, PLR, NLR, PPV, and NPV, using microscopy as the gold standard. Indeed, *Pv18SrRNA* showed a Se of 93.65% and Sp of 43.75%, a PPV of 86.76% and an NPV of 63.64% at detecting all stages. In contrast, no significant differences in Se and Sp for detection of gametocytes was found between, *PvLAP5* (Se of 94.29% and Sp of 31.82%) and *Pvs25* (Se of 100.00% and Sp of 29.55%), nor in the PPV, or the probability of detecting a true positive gametocyte sample (PPV = 52.38 and 53.03% respectively), but *Pvs25* showed a higher NPV compared to *PvLAP5* (100% vs 87.50%) (**Table 4 and Table G in S1 File**).

**Table 4:** Summary results of the field performance of a qRT-PCR assay for the detection of Plasmodium vivax gene transcripts in field samples preserved at ambient temperature in RNAprotect compared to microscopy.

					Assays performance results								
Target	Assay	Se	(95% CI)	Sp	(95% CI)	PLR	(95% CI)	NLR	(95% CI)	PPV	(95% CI)	NPV	(95% CI)
*P*. *vivax (all stages)*	*18SrRNA*	93.65	(84.53, 98.24)	43.75	(19.75, 70.12)	1.66	(1.08, 2.58)	0.15	(0.05, 0.44)	86.76	(80.90, 91.03)	63.64	(36.83, 84.01)
*P*. *vivax* (gametocytes)	*Pvs25*	100.00	(90.00, 100.00)	29.55	(16.76, 45.20)	1.42	(1.17, 1.72)	0	-	53.03	(48.25, 57.75)	100	-
	*LAP5*	94.29	(80.84, 99.30)	31.82	(18.61, 47.58)	1.38	(1.11, 1.72)	0.18	(0.04, 0.74)	51.38	(46.95, 57.76)	87.5	(63, 96.64)

Se = Sensitivity

Sp = Specificity

PLR = Positive Likelihood Ratio

NLR = Negative Likelihood Ratio

PPV = Positive predictive value

NPV = Negative predictive value

## Discussion

As malaria continues to decline [14], elimination from residual foci with persisting transmission represents a major barrier in countries approaching malaria elimination [12,57]. To closely monitor advances towards elimination, it is important to maintain robust malaria molecular epidemiological surveillance programs, especially in remote areas that lack the infrastructure to maintain a cold chain.

It is known that *P*. *vivax* gametocytes are present in the circulation even before the onset of symptoms and that these positively mirror asexual parasitemias [35], the recent description of major cryptic reservoirs of *P*. *vivax* blood stages in the spleen [23] and the extravascular spaces of the bone marrow [21] might help explain why - apart from relapses caused by the hypnozoites, *P*. *vivax* remains endemic and more difficult to eliminate than *P*. *falciparum*.

*P*. *vivax* gametocytes represent a small fraction of the total parasite mass found in an infected individual, especially in asymptomatic patients with generally low parasite load. Consequently, detection and quantification of *P*. *vivax* gametocytes by LM has limited application for the determination of the transmission reservoir, this that presents a challenge to the microscopist. Therefore, molecular diagnostic methods are an important alternative in malaria elimination programs to more accurately determine the transmission reservoir and design interventions tailored to endemic areas of low transmission [44,58].

*P*. *vivax* qRT-PCR assays based on detection of *Pv18SrRNA* and *Pvs25* from low blood volume field samples stored at ambient temperature, have been previously validated for molecular epidemiological studies [27,37]. The method takes advantage of abundant *Pv18SrRNA* transcripts present in circulating *P*. *vivax* blood stage parasites. Similar approaches for *P*. *vivax* gametocyte detection by qRT-PCR using *Pvs25* have been described [34,37,58,59]. We have previously demonstrated that *PvLAP5* detects *P*. *vivax* gametocytes in tissues from experimentally infected NHPs, both using specific antibodies and by qRT-PCR [21]. Unlik*e Pvs25*, the *PvLAP5* qRT-PCR detection assay uses exon-spanning primers, thereby minimising spurious amplification from gDNA.

In this study we compared the performance of a *PvLAP5* qRT-PCR gametocyte detection assay to detection by LM and the gold standard gametocyte molecular marker *Pvs25* [37], using clinical field samples preserved in RNAp at ambient temperature from remote areas of Panama.

First, we demonstrate that at least 60 μL of peripheral blood were sufficient for the detection of *PvLAP*5 and *Pv18SrRNA* and no significant differences were found in the mean Ct values between blood kept at room temperature in RNAp for 8 days and frozen until process, or snap-frozen at -80° C upon collection, using experimentally infected *P*. *vivax Aotus* monkey blood. Although we did not specifically test the quality of the RNA in this experiment, the difference in the Ct values of *Pv18SrRNA* and *PvLAP5* between conditions indicated a lower amount of RNA in the samples kept at room temperature, suggesting degradation of RNA over time, as it has been shown by others [60]. Furthermore, our findings indicate that 59/63 (93.6%) of the field samples examined tested positive for *Pv18SrRNA* after spending a median of 7 (0, 192) days in transit. This observation supports the suitability of RNAp to preserve *P*. *vivax* samples at ambient temperature for prolonged periods of time, as it has been demonstrated in previous studies with low blood volume samples preserved in filter paper [61], RNA stabilization buffer [46] or RNAp [58,59,62].

Major challenges for preservation of RNA samples in the field, when collection sites were in remote areas and difficult to access (only by plane or boat) included: i) the time between the collection of the sample and its arrival at the laboratory, and ii) loss of the sample volume due to evaporation or leakage attributed to loose caps or atmospheric pressure changes encountered during transport by plane to the laboratory. However, the consistency of *Pv18SrRNA* gene expression across samples suggests that despite the difficulties encountered, the overall gene expression pattern of the markers examined was not dramatically affected.

We had previously determined the analytical sensitivity of the *PvLAP5* qRT-PCR assay [21]. In this study we used a serial dilution of a known *P*. *vivax* sample and determined the LOD of the *PvLAP5* and *Pvs25* qRT-PCR assays to be 5 to 50-fold more sensitive respectively than the theoretical LOD of 9.6 gametocytes x μL reported for the *Pvs25* qRT-PCR assay by others [55]. On the other hand, LM detected *P*. *vivax* gametocytes in 55.5% of the positive smears examined, 17 to 34% less than what had been reported in a large scale epidemiological survey carried out in Indonesia and Thailand, where gametocytes detection by LM fluctuated between 66.6% and 84.3% at enrolment respectively [35]. Likewise, in a longitudinal study done in two regions of Peru, gametocytes were present in as low as 28.4% of *P*. *vivax* infections with a peak of 61.5% at the start of the transmission season [62]. Similar observations were made in a recent study from Brazil, Thailand, Papua New Guinea, and the Solomon Islands, were *P*. *vivax* gametocytes were detected in 23–72% of the samples examined [58].

Using gametocyte markers *PvLAP5* and *Pvs25*, we were able to detect *P*. *vivax* gametocytes gene transcripts in 88.8% and 90.4% of the samples examined irrespective of their parasitemia level. These high detection rates contrast with those of other studies using a *Pvs25* qRT-PCR assay that reported a detection rate as low as 23.5% in the Solomon islands in 2012, and 49% in a study from Papua New Guinea in 2010. Although, rates up to 96% have been observed in a small cohort study from Brazil [29]. Other studies have reported gametocyte detection rates of 69% using *Pvs25* qRT-PCR in clinical samples positive for Pv18SrRNA [37]. Such variability in detection rates by the *Pvs25* qRT-PCR assay has been attributed to endemicity level and age of subjects [58], differences in study design, seasonality of transmission [29], parasitemia level and the amount of blood examined [63].

The high molecular gametocyte detection rate found in this study indicates that almost every *P*. *vivax* infection carried mature gametocytes and had the potential to transmit parasites at the time of the survey. The observation that both gametocytes markers were strongly correlated to parasitemia and the constitutive marker *Pv18SrRNA*, and that gametocytemia closely mirror asexual parasitemia, suggests that *Pv18SrRNA* may be an accurate predictor of *P*. *vivax* gametocyte carriage as it has been observed by others [58].

Finally, no statistically significant differences were detected in the Se (100% vs 94.29%), Sp (29.5% vs 31.28%) and PPV (53.03% vs 52.38%) between the *Pvs25* and *PvLAP5* assays, yet *Pvs25* showed an NPV of 100% vs 87.50% for *PvLAP5*, indicating the suitability of the latter as a gametocyte detection assay.

Taken together, the data of this study suggest that *PvLAP5* is as sensitive and specific as *Pvs25* at detecting *P*. *vivax* gametocytes by qRT-PCR in low blood volume field samples preserved in RNAp at ambient temperature. Moreover, the strong correlation found in this study between gametocyte markers *Pvs2*5 and *PvLAP5* confirmed our hypothesis that *PvLAP5* is a suitable marker to monitor sexual parasitemias that could be useful to inform small scale spatial variability in transmission intensity as it has been suggested for others molecular markers [45]. Hence, the *PvLAP5* qRT-PCR assay should be considered a suitable assay for the determination of the human transmission reservoir in malaria molecular epidemiological surveys [37].

Interestingly, during 2019-2020, a twofold increase in malaria cases compared to the previous two years was observed in Panama, suggesting that an epidemic outbreak was occurring at the time. Indeed, in 2021 Panama registered 4,121 malaria cases, up 2.2 times compared to 2020 [64]. Reasons for this outbreak are currently unclear but in past epidemic outbreaks of malaria these have been associated with extreme weather events such as tropical storms and hurricanes [65], particularly those that affected Central America and the Caribbean during 2019-2020 [53]. For instance, hurricanes, and other extreme weather events such as the “El Niño Southern Oscillation (ENSO)”, which was particularly strong during 2018-2019 in the region [66], have been associated with changes in malaria transmission in Panama and the Caribbean [5]. However, we cannot rule out that other factors, such as increased case detection due to the introduction of Malaria RDTs by the Panamanian Ministry of Health in 2017 [1], changes in vectorial behaviour [67] and transmission efficiency [68,69], reintroduction of parasites [4,6], waning immunity due to lack of exposure [70], socioeconomic factors [8], or prolonged confinement experienced by the population for the control of COVID-19 during 2020, might have all or in part, contributed to its development.

In a previous *P*. *vivax* population genomic study in Panama [6], we observed low diversity among *P*. *vivax* parasites indicative that this population had recently been subjected to a severe bottleneck. Hence, at the time (2009-2019), we postulated that malaria in Panama was amenable to elimination. We also identified imported *P*. *vivax* parasites from diverse geographic locations, which did not appear to have influenced the overall parasite population structure at the time, suggesting that transmission from such cases was limited and did not pose a major impediment to elimination. Simultaneous with the malaria epidemic of 2021 [64], there was an unprecedented transit of 136,000 continental and extracontinental migrants that crossed the Darién Gap from South America. This number is higher than the previous 11 years combined (from 2009 to 2020), when 117,887 migrants crossed the isthmus heading north [54]. Such large numbers of transit migrants over a short period of time, combined with COVID-19-related reduction in surveillance activities, altogether represent a significant setback at achieving the goal of eliminating malaria from Panama by 2025 [71]. Therefore, it might be important to consider including qRT-PCR assays for detection of asexual and sexual *P*. *vivax* stages as part of the screening process to determine the hidden reservoir of malaria transmission among the transit migrants in Darien, as it has been implemented elsewhere [72].

Lastly, it is worth stating that the use of qRT-PCR for the detection of *P*. *vivax* gametocytes is not yet a cost-effective option to be implemented as part of a national malaria elimination campaign to replace LM in Panama. Nevertheless, as the elimination campaign progresses and with fewer cases and well-trained microscopists available, the utility of LM decreases. Hence, in such low transmission scenarios, qRT-PCR will become very useful to determine the prevalence of gametocyte carriage and to quantify the hidden reservoir of transmission across endemic regions.

## Supporting information

S1 File **Fig A: Epidemiology of *Plasmodium vivax* malaria in Panama between 2017-2020.** a) Map of Panama showing the incidence of *P*. *vivax* cases by 10,000 population at the corregimiento level for years 2017-2020. b) Number of *P*. *vivax* cases per year. c) Number of *P*. *vivax* cases stratified by age for years 2017-2020. d) Percentage of *P*. *vivax* cases stratified by race and ethnicity for years 2017-2019. Base map downloaded from www.gadm.org under licence CC-BY. www.gadm.org/maps/PAN_1.html. **Fig B: Typical amplification and melt curves plots of a qRT-PCR assay for the detection of gametocyte stage-specific markers PVX_111175 (*Pvs25*), PVX_117900 (*PvLAP5*) and constitutive gene *Pv18SrRNA***. a) Amplification curves plot of *P*. *vivax* positive human controls B and S; b) Melt curves plot of positive *P*. *vivax* human controls B and S. Each assay was run in triplicate. **Fig C: Optimization of blood volume and sample preservation conditions for detection of *P*. *vivax* by qRT-PCR**. Gene expression Ct values of gametocyte stage-specific markers *PvLAP5* and constitutive gene *Pv18SrRNA* qRT-PCR assays using 60 or 120 μL of *Aotus P*. *vivax* SAL-1 infected blood preserved in 500 μL of RNAp under different environmental conditions. Parasitemia of *Aotus* blood donor 51,080 parasites x μL. ns = non-significant (two-way ANOVA). **Fig D: Melt curves plots of a qRT-PCR assay for the detection of gametocyte stage-specific markers PVX_117900 (*PvLAP5*) and PVX_111175 (*Pvs25*) in malaria naïve lab-bred *Aotus* monkeys use as negative controls**. Melt curves plots showing non-specific products (primer dimers) (arrow heads) in monkeys MN28030, MN31012, MN33036 and MN29002. Arrows show positive controls: *PvLAP5* and *Pvs25*. **Fig E: Melt curves plots of a qRT-PCR assay for the detection of gametocyte stage-specific markers PVX_117900 (*PvLAP5*) and PVX_111175 (*Pvs25*) and Pv18SrRNA in healthy malaria negative controls**. Melt curves plots showing non-specific products (primer dimers) (arrow heads) from six selected samples for illustration. Arrows show positive controls: a–b) *PvLAP5*; c-d) *Pvs25*; e-f) *Pv18SrRNA*. **Table A**: Primer sequences of *Plasmodium vivax* constitutive and gametocyte stage specific markers of a qRT-PCR assay. **Table B**: Epidemiology and qRT-PCR data of *Plasmodium vivax* constitutive marker *Pv18SrRNA* and gametocyte specific genes PVX_111175 (*Pvs25*) and PVX_117900 (*PvLAP5*) from microscopic negative controls collected in Panama during 2017-2019. **Table C**: *Plasmodium vivax* qRT-PCR data Ct values for constitutive marker *Pv18SrRNA* and gametocyte specific genes PVX_111175 (*Pvs25*) and PVX_117900 (*PvLAP5*) from malaria naïve *Aotus* monkeys. **Table D**: Frequency distribution of *Plasmodium vivax* samples collection sites stratified by province and district for years 2017-2020. **Table E**: Demographic and socioeconomic characteristics of selected study participants for the validation of a qRT-PCR assay for the detection of *Plasmodium vivax* gametocytes in field isolates collected from Panama during 2017-2020. **Table F**: Epidemiologic and qRT-PCR data of *Plasmodium vivax* constitutive marker *Pv18SrRNA* and gametocyte specific genes *Pvs25* and *PvLAP5* from microscopic positive field isolates collected in Panama during 2017-2020. **Table G**: Field validation of a qRT-PCR assay for the detection of *Plasmodium vivax* gene transcripts in smear positive and negative field samples preserved at ambient temperature in RNAprotect compared against microscopy.
(PDF)
