# Supplementary material for: Comparison of PvLAP5 and Pvs25 qRT-PCR assays for the detection of Plasmodium vivax gametocytes in field samples preserved at ambient temperature from remote malaria endemic regions of Panama
Source: PLoS Negl Trop Dis. 2022 Apr 8;16(4):e0010327. doi: 10.1371/journal.pntd.0010327 (PMC9020738; doi:10.1371/journal.pntd.0010327)
Supplement: S1 File — Fig A: Epidemiology of Plasmodium vivax malaria in Panama between 2017-2020. a) Map of Panama showing the incidence of P. vivax cases by 10,000 population at the corregimiento level for years 2017-2020. b) Number of P. vivax cases per year. c) Number of P. vivax cases stratified by age for years 2017-2020. d) Percentage of P. vivax cases stratified by race and ethnicity for years 2017-2019. Base map downloaded from www.gadm.org under licence CC-BY. www.gadm.org/maps/PAN_1.html. Fig B: Typical amplification and melt curves plots of a qRT-PCR assay for the detection of gametocyte stage-specific markers PVX_111175 (Pvs25), PVX_117900 (PvLAP5) and constitutive gene Pv18SrRNA. a) Amplification curves plot of P. vivax positive human controls B and S; b) Melt curves plot of positive P. vivax human controls B and S. Each assay was run in triplicate. Fig C: Optimization of blood volume and sample preservation conditions for detection of P. vivax by qRT-PCR. Gene expression Ct values of gametocyte stage-specific markers PvLAP5 and constitutive gene Pv18SrRNA qRT-PCR assays using 60 or 120 μL of Aotus P. vivax SAL-1 infected blood preserved in 500 μL of RNAp under different environmental conditions. Parasitemia of Aotus blood donor 51,080 parasites x μL. ns = non-significant (two-way ANOVA). Fig D: Melt curves plots of a qRT-PCR assay for the detection of gametocyte stage-specific markers PVX_117900 (PvLAP5) and PVX_111175 (Pvs25) in malaria naïve lab-bred Aotus monkeys use as negative controls. Melt curves plots showing non-specific products (primer dimers) (arrow heads) in monkeys MN28030, MN31012, MN33036 and MN29002. Arrows show positive controls: PvLAP5 and Pvs25. Fig E: Melt curves plots of a qRT-PCR assay for the detection of gametocyte stage-specific markers PVX_117900 (PvLAP5) and PVX_111175 (Pvs25) and Pv18SrRNA in healthy malaria negative controls. Melt curves plots showing non-specific products (primer dimers) (arrow heads) from six selected samples for illustratio [file pntd.0010327.s001.pdf]

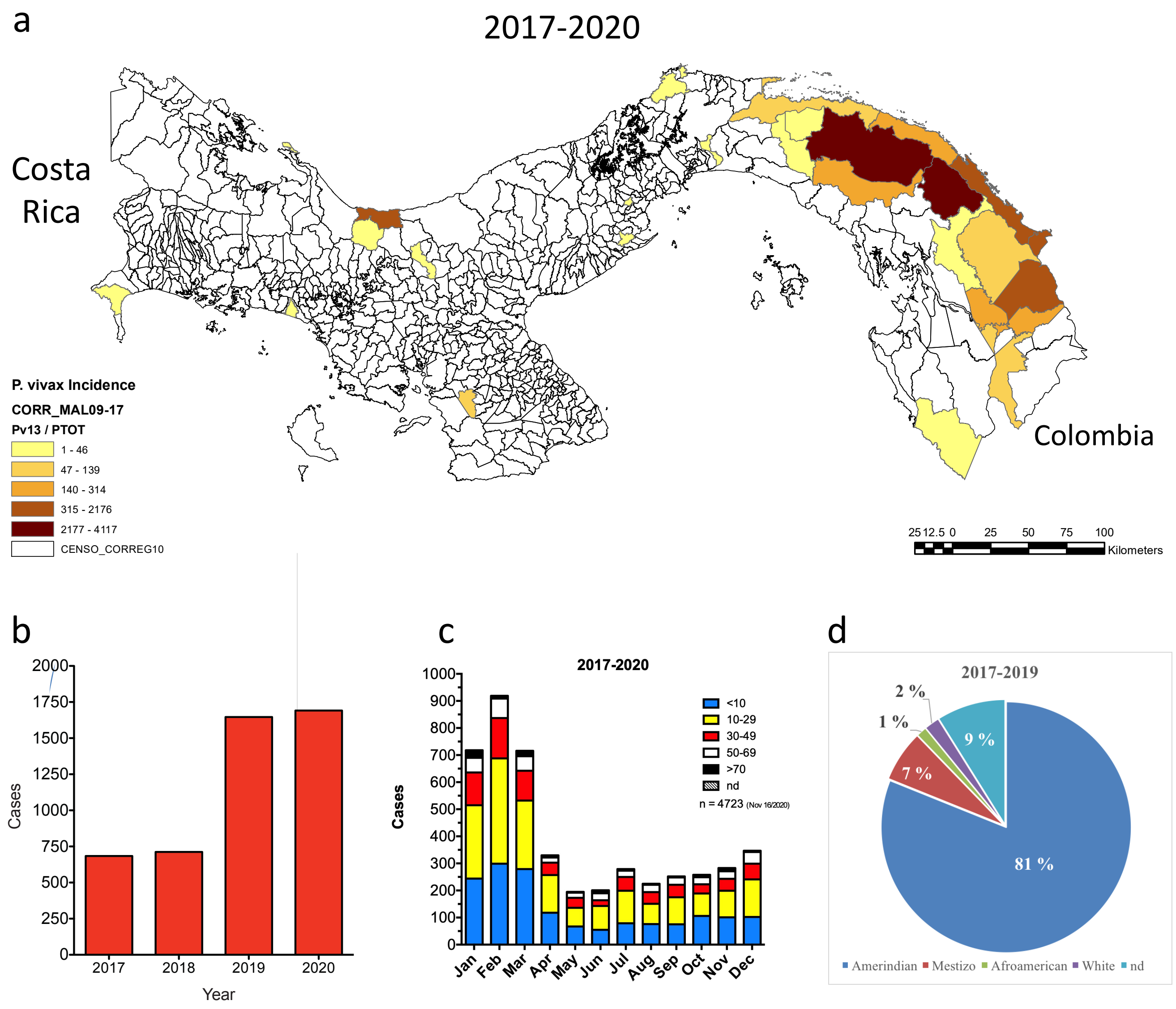

Fig A. Epidemiology of *Plasmodium vivax* malaria in Panama between 2017-2020. a) Map of Panama showing the incidence of *P. vivax* cases by 10,000 population at the corregimiento level for years 2017-2020. b) Number of *P. vivax* cases per year. c) Number of P. vivax cases stratified by age for years 2017-2020. d) Percentage of *P. vivax* cases stratified by race and ethnicity for years 2017-2019. Base map downloaded from www.gadm.org under licence CC-BY. www.gadm.org/maps/PAN_1.html

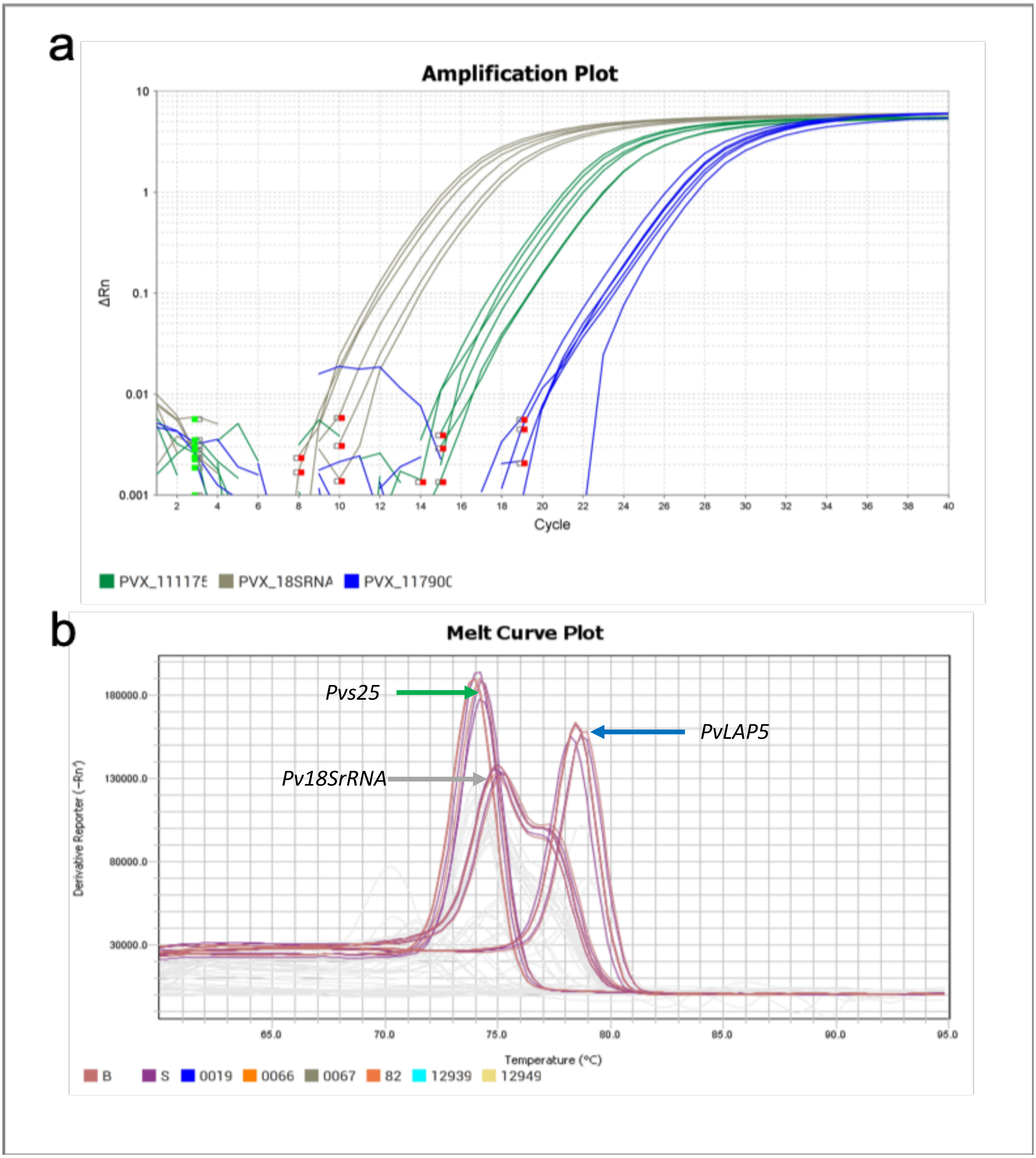

Fig B. Typical amplification and melt curves plots of a qRT-PCR assay for the detection of gametocyte stage-specific markers PVX_111175 (*Pvs25*), PVX_117900 (*PvLAP5*) and constitutive gene *Pv18SrRNA*. a) Amplification curves plot of *P. vivax* positive human controls B and S; b) Melt curves plot of positive *P. vivax* human controls B and S. Each assay was run in triplicate.

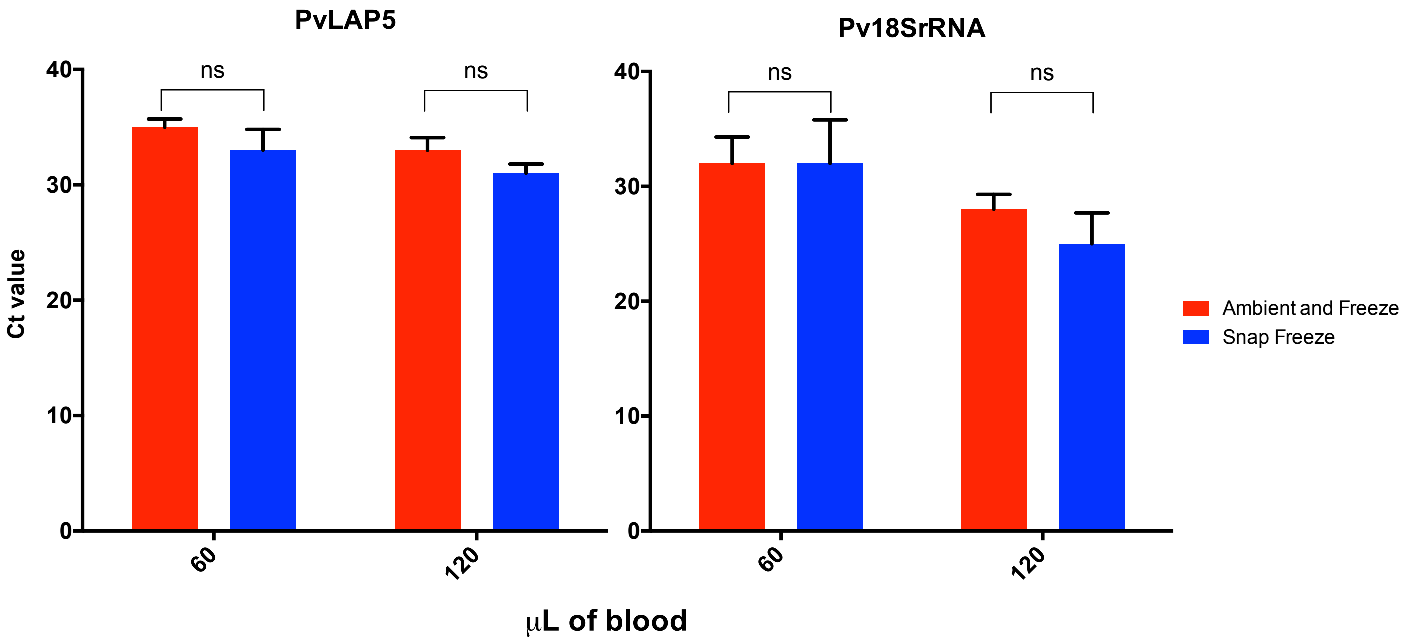

Fig C. Optimization of blood volume and sample preservation conditions for detection of *P. vivax* by qRT-PCR. Gene expression Ct values of gametocyte stage-specific markers *PvLAP5* and constitutive gene *Pv18SrRNA* qRT-PCR assays using 60 or 120 µL of Aotus *P. vivax* SAL-1 infected blood preserved in 500 µL of RNAp under different environmental conditions. Parasitemia of Aotus blood donor 51,080 parasites x µL. ns = non-significant (two-way ANOVA).

Table A. Primer sequences of *Plasmodium vivax* constitutive and gametocyte stage specific markers of a qRT-PCR assay.

Gene Name	Gene ID	Chromosome	Pf ortholog	Primer sequence (5'-3')	Primer efficiency
LCCL domain-containing protein (LAP5)	PVX_117900	12	PF3D7_1451600 (LCCL domain-containing protein, LAP5)	CGCGCGTTTTGTAGGGAGCC<br>GGCGGTACTCCGTCAGTTTCTTCA	94.82%
Ookinete surface protein (Pvs25)	PVX_111175	6	PF3D7_1031000 (25 kDa ookinete surface antigen precursor, Pfs25)	GGCAAAGTCCCCAATCCAGA<br>GCCTTCCATACACTGGCACT	97.99%
Pv18SrRNA			-	GCTTTGTAATTGGAATGATGGGAAT<br>ATGCGCACAAAGTCGATACGAAG	113.60%

Table B. Epidemiologic and qRT-PCR data of *Plasmodium vivax* constitutive marker Pv18SrRNA and gametocyte specific genes PVX_111175 (*Pvs25*) and PVX_117900 (*PvLAP5*) from microscopic negative controls collected in Panama during 2017-2019.

Id	Code	Gender	Age	Province	Days	Parasitemia %	PvLAP5	Pvs25	Pv18SrRNA	Result
1	C18001	M	59	Veraguas	0	Neg.	37	40	36	0
2	C18002	M	56	Cocle	0	Neg.	35	37	36	0
3	C18003	M	38	Guna Yala	0	Neg.	38	36	40	0
4	C18004	M	63	Veraguas	0	Neg.	40	37	40	0
5	C18005	M	59	Panama	1	Neg.	37	40.	37	0
6	C19006	M	nd	Darien	1	Neg.	40	40	40	0
7	C19007	M	52	Panama	1	Neg.	36	35	37	0
8	C19008	M	65	Panama	1	Neg.	40	40	40	0
9	C19009	F	nd	Panama	1	Neg.	36	38	40	0
10	C19010	F	nd	Panama	1	Neg.	40	40	36	0
11	C19011	F	24	Panama	1	Neg.	36	36	37	0
12	C19012	M	32	Panama	0	Neg.	40	40	36	0
13	C19013	F	51	Panama	0	Neg.	40	37	36	0
14	C19014	M	29	Panama	0	Neg.	40	37	40	0
15	C19015	M	37	Panama	0	Neg.	40	37	40	0
16	C19016	M	62	Panama	0	Neg.	40	40	38	0
Median (range)			52 (24, 65)		0(0, 1)	GM (95% CI)	38.39 (37.36, 39.45)	38.08 (37.12, 39.01)	38.02 (37.05, 39.01)	

Table C. *Plasmodium vivax* qRT-PCR data Ct values for constitutive marker Pv18SrRNA and gametocyte specific genes PVX_111175 (*Pvs25*) and PVX_117900 (*PvLAP5*) from malaria naive Aotus monkeys.

Id	Monkey	Sex	Parasitemia %	*PvLAP5*	*Pvs25*	*Pv18SrRNA*	Result
1	24004	F	Neg.	40	40	40	0
2	27035	M	Neg.	40	40	40	0
3	28030	M	Neg.	35	40	40	0
4	29002	M	Neg.	40	35	40	0
5	29010	F	Neg.	40	40	40	0
6	30027	F	Neg.	40	40	40	0
7	31012	F	Neg.	34	40	40	0
8	31037	M	Neg.	40	40	40	0
9	33032	M	Neg.	40	40	40	0
10	33036	M	Neg.	36	40	40	0
11	33038	F	Neg.	40	40	40	0
12	33039	M	Neg.	40	40	40	0
13	33045	M	Neg.	40	40	40	0
14	33051	F	Neg.	40	40	40	0
15	33053	F	Neg.	40	40	40	0
	Mean		GM (95% CI)	38.94 (37.75, 40.18)	39.65 (38.90, 40.41)	40	

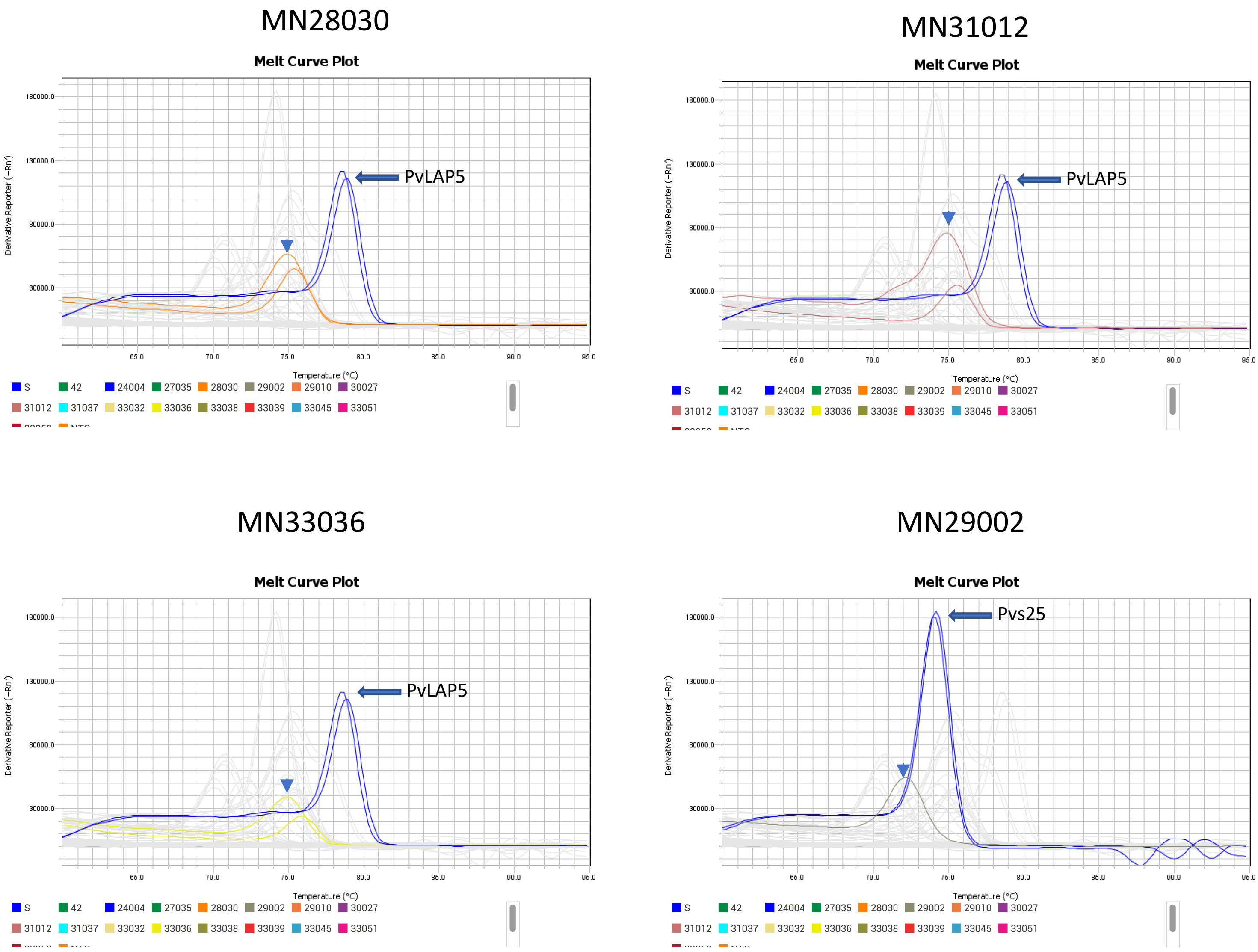

Fig D. Melt curves plots of a qRT-PCR assay for the detection of gametocyte stage-specific markers PVX_117900 (*PvLAP5*) and PVX_111175 (*Pvs25*) in malaria naïve lab-bred Aotus monkeys use as negative controls. Melt curves plots showing non-specific products (primer dimers) (arrow heads) in monkeys MN28030, MN31012, MN33036 and MN29002. Arrows show positive controls: PvLAP5and Pvs25.

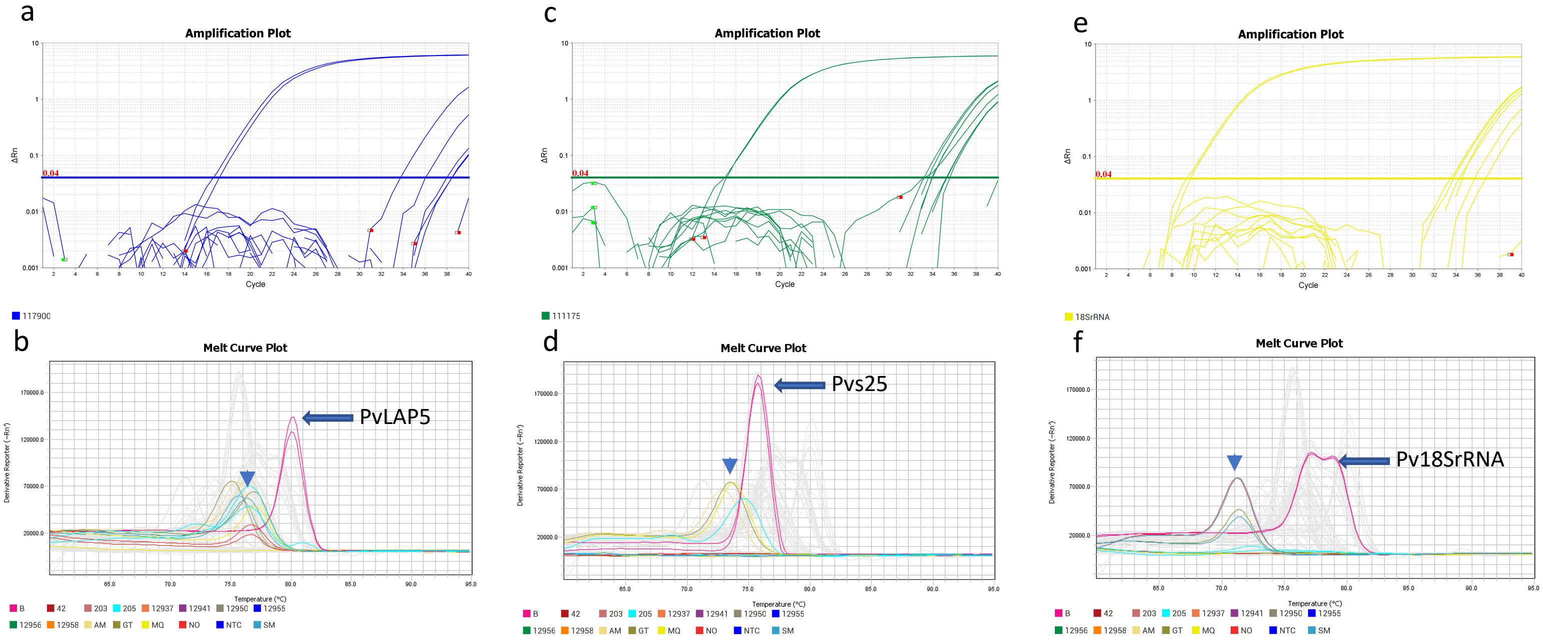

Fig E. Melt curves plots of a qRT-PCR assay for the detection of gametocyte stage-specific markers PVX_117900 (*PvLAP5*) and PVX_111175 (*Pvs25*) and Pv18SrRNA in healthy malaria negative controls. Melt curves plots showing non-specific products (primer dimers) (arrow heads) from six selected samples for illustration. Arrows show positive controls: a–b) PvLAP5; c-d) Pvs25; e-f) Pv18SrRNA.

Table D. Frequency distribution of *Plasmodium vivax* samples collection sites stratified by province and district for years 2017-2020.

Sample collection site			
Province	District	Frequency	(%)
Darién	Cemaco	6	(10)
	Cirilo Guainora	1	(2)
	Manuel Ortega	9	(14)
	Pinogana	10	(16)
Panama	Arraijan	1	(2)
	Chepo	20	(32)
	La Chorrera	1	(2)
	Panamá	2	(3)
Guna Yala	Tubuala	13	(21)
Total		63	(100)

Table E. Demographic and socioeconomic characteristics of selected study participants for the validation of a qRT-PCR assay for the detection of *Plasmodium vivax* gametocytes in field isolates collected from Panama during 2017-2020.

		Frequency	%
**Gender**			
	Male	47	64
	Female	26	36
Total		73	100
**Race / Ethnicity**			
	Amerindian	31	42
	Mestizo	7	10
	Afro	1	1
	White	2	3
	Other	1	1
	nr	31	42
Total		73	100
**Place of residence**			
	Cocle	1	1
	Darien	19	26
	Guna Yala	20	27
	Panama	29	40
	Veraguas	2	3
	nr	2	3
Total		73	100
**Employment**			
	Yes	8	11
	No	19	26
	nr	46	63
Total		73	100
**Literacy**			
	Yes	30	41
	No	10	14
	nr	33	45
Total		73	
**House Type**			
	1	2	3
	2	16	22
	3	16	22
	4	1	1
	5	1	1
	nr	37	51
Total		73	100

n = 73 survey participants

nr = non-responders

Table F. Epidemiologic and qRT-PCR data of *Plasmodium vivax* constitutive marker *Pv18SrRNA* and gametocyte specific genes *Pvs25* and *PvLAP5* from microscopic positive field isolates collected in Panama during 2017-2020.

No.	Code	Gametocytes	Province	Gender	Age	Days	RNA	Parasitemia`%	PVX_117900	PVX_111175	PVX_18sRNA
1	17001	1	Panama	F	3	5	1	0.26	34	32	33
2	18002	0	Panama	M	5	7	35.4	0.25	32	32	38
3	18003	1	Panama	F	3	7	17.8	0.1	40	33	32
4	18004	1	Panama	F	2	7	16.5	0.34	35	31	34
5	18005	1	Panama	F	0.5	2	32.7	0.05	33	31	36
6	18006	0	Panama	M	2	10	1.3	0.11	34	33	33
7	18007	0	Panama	M	nd	2	1.4	0.25	38	40	35
8	18008	0	Guna Yala	M	18	2	25.9	0.16	33	40	35
9	18009	1	Panama	M	nd	1	10.1	0.37	30	26	18
10	19010	0	Panama	F	nd	1	7.2	0.2	31	26	19
11	19012	0	Guna Yala	F	nd	40	31.9	0.23	36	36	32
12	19013	0	Guna Yala	M	nd	38	8.6	0.24	35	36	27
13	19014	1	Guna Yala	F	nd	38	59.4	0.12	37	37	30
14	19016	0	Guna Yala	M	nd	36	6.3	0.05	38	34	26
15	19017	1	Guna Yala	M	19	31	18.6	0.15	32	31	25
16	19020	1	Panama	M	34	7	5.9	0.66	33	30	22
17	19021	0	Panama	M	33	8	-0.9	0.14	32	29	20
18	19022	1	Panama	F	nd	1	1.6	0.13	32	29	28
19	19025	0	Panama	M	14	4	49.9	0.88	35	34	28
20	19027	1	Panama	M	nd	0	16.1	0.48	33	33	30
21	19028	0	Panama	F	nd	0	15	0.18	37	37	34
22	19029	0	Panama	F	nd	3	7.2	0.15	33	30	22
23	19030	0	Guna Yala	F	29	2	34.1	0.42	40	38	37
24	19031	1	Panama	M	24	3	6.5	0.09	28	26	18
25	19032	1	Panama	F	2	5	3.4	0.35	30	28	20
26	19033	0	Panama	F	53	14	3.9	0.25	40	33	28
27	19034	1	Panama	M	nd	1	13.6	0.38	36	31	22
28	19036	1	Darien	M	15	4	11.1	0.62	31	27	16
29	19039	1	Guna Yala	F	25	6	1.7	0.4	40	32	36
30	19040	1	Guna Yala	M	5	4	17.3	0.82	33	31	29
31	19041	1	Darien	M	27	4	-0.6	0.09	36	33	22
32	19042	0	Darien	F	44	4	4	0.16	37	40	37
33	19043	1	Darien	M	42	1	8.9	0.001	29	25	15
34	19044	1	Darien	M	76	1	20.8	1.35	26	25	21
35	19045	1	Panama	M	71	11	7.6	0.4	35	31	21
36	19046	0	Panama	M	19	11	19.6	0.16	40	33	24
37	19047	1	Guna Yala	M	38	7	4.9	0.28	32	28	20
38	19048	0	Darien	F	0.6	14	2.8	0.08	37	40	38
39	19050	1	Guna Yala	M	7	4	6.4	0.95	33	30	26
40	19051	1	Guna Yala	M	16	8	2.3	0.38	35	36	28
41	19052	1	Guna Yala	M	28	4	3.2	0.32	32	29	25
42	19058	1	Panama	M	50	0	6.4	0.47	31	30	26
43	19059	1	Darien	M	2	7	0	1.04	29	27	21
44	19063	1	Darien	M	58	7	2.8	0.13	31	36	40
45	19064	0	Darien	F	65	3	3.6	0.04	31	30	29
46	19065	1	Darien	F	56	13	1.3	0.75	37	35	37
47	19066	1	Darien	M	51	6	8.7	0.63	34	36	36
48	19067	0	Darien	M	55	9	2.6	0.04	35	32	31
49	19068	0	Darien	F	74	31	-0.5	0.18	34	40	40
50	19069	0	Darien	F	49	16	1.2	0.05	35	35	32
51	19070	0	Panama	M	19	6	nd	0.14	29	28	24
52	19071	0	Darien	F	10	10	3	0.07	40	35	29
53	19072	1	Darien	M	56	6	2	0.06	37	34	32
54	19073	0	Darien	M	55	14	22.9	0.04	38	36	40
55	19075	0	Darien	M	45	6	1.1	0.11	35	40	32
56	19076	0	Darien	F	6	12	1	0.05	40	35	29
57	19077	1	Darien	M	6	12	-0.8	0.29	33	33	26
58	19078	1	Darien	M	12	11	1.8	0.66	31	29	24
59	19079	1	Darien	M	39	7	2.2	0.05	34	31	26
60	19081	1	Darien	M	nd	nd	1.2	0.35	30	28	21
61	19082	0	Darien	F	19	192	6.3	0.11	34	36	33
62	19083	0	Darien	F	49	192	4.5	1.39	31	34	32
63	19084	1	Darien	M	69	192	6	0.21	32	35	40
**Median**					**26**	**7**					
**Min**					0.5	0					
**Max**					76	192					

Ct values

15 40

Table G. Field validation of a qRT-PCR assay for the detection of *Plasmodium vivax* gene transcripts in smear positive and negative field samples preserved at ambient temperature in RNAprotect compared against microscopy.

Evaluation											
Pv18SrRNA				Pvs25				PvLAP5			
All stages	Positive	Negative	Total	Gametocytes	Positive	Negative	Total	Gametocytes	Positive	Negative	Total
Microscopy Positive	59	9	68	Positive	35	31	66	Positive	33	30	63
Microscopy Negative	4	7	11	Negative	0	13	13	Negative	2	14	16
Total	63	16	79	Total	35	44	79	Total	35	44	79

	Pv18SrRNA %	Pv18SrRNA 95 % CI	Pvs25 %	Pvs25 95 % CI	PvLAP5 %	PvLAP5 95 % CI
Sensitivity	93.65	84.53, 98.24	100.00	90.00, 100.00	94.29	80.84, 99.30
Specificity	43.75	19.75, 70.12	29.55	16.76, 45.20	31.82	18,61, 47.58
PLR	1.66	1.08, 2.58	1.42	1.17, 1.72	1.38	1.11, 1.72
NLR	0.15	0.05, 0.44	0.00	-	0.18	0.04, 0.74
PPV	86.76	80.90, 91.03	53.03	48.25, 57.75	52.38	46.94, 56.76
NPV	63.64	36.83, 84.01	100.00	-	87.50	63.00, 96.64

PLR = Positive Likelihood Ratio
NLR = Negative Likelihood Ratio
PPV = Positive Predictive Value
NPV = Negative Predictive Value
